# Associations among working hours, sleep duration, self-rated health, and health-related quality of life in Korean men

**DOI:** 10.1186/s12955-020-01538-2

**Published:** 2020-08-24

**Authors:** Darae Woo, Yeonjin Lee, Sangshin Park

**Affiliations:** 1 Graduate School of Urban Public Health, University of Seoul, 163 Seoulsiripdae-ro, Dongdaemun-gu, Seoul, 02504 Republic of Korea; 2 Incheon Metropolitan City Public Health Policy Institute, Incheon, Republic of Korea; 3 Department of Social Work and Social Administration, The University of Hong Kong, Hong Kong, Hong Kong; 4 School of Public Health, LKS Faculty of Medicine, The University of Hong Kong, Hong Kong, Hong Kong

**Keywords:** Working hours, Health-related quality of life, Sleep duration, Self-rated health, Men

## Abstract

**Background:**

This study aimed to examine the relationship between working hours and health-related quality of life (HRQOL) in men and to determine whether this relationship was mediated by sleep duration and self-rated health (SRH).

**Materials and methods:**

Our study population included 2141 working men aged 20 to 49 years old from the Korea National Health and Nutrition Examination Survey 2016–2017. Working hours, SRH, and HRQOL were measured by a structured questionnaire survey. Sleep duration was calculated based on self-reported data. We investigated the association between working hours and HRQOL and performed a mediation analysis to evaluate the contributions of sleep duration and poor SRH to this relationship.

**Results:**

This study identified a significant association between working hours and HRQOL. Long working hours directly and indirectly affected HRQOL through poor SRH. Men who overworked were more likely to report poor HRQOL due to worsened SRH. Poor SRH was responsible for 26.3% of this relationship. But sleep duration did not explain the relationship between working hours and HRQOL.

**Conclusions:**

Working hours were directly associated with HRQOL in men. Furthermore, there was an indirect effect of working hours on HRQOL mediated by poorer SRH. An improved work-life balance is necessary to promote men’s health and quality of life.

## Introduction

Korea has a long history of a male-dominated labor market. Korean men are responsible for their livelihood as the head of the household, and household duties are considered the primary responsibilities of women [1]. In 2018, the labor force participation rate was 73.7% in men while 52.9% in women [2]. Many Korean firms and organizations have masculine cultures, hierarchical structures, and normative rules that value longer working hours [3]. Collective practices rooted in organizations cause Korean male workers, who are generally single bread-winners, to have longer average working hours than their counterparts in other countries [4].

Although the International Labour Organization has recommended that maximum working hours should not exceed 40 h per week [5], Korean male workers work more than 45.5 h per week on average, whereas countries in the Organization for Economic Cooperation and Development (OECD) reported an average of 42.3 working hours per week in 2018 [6]. To improve work-life balance, the Korean Labor Standards Act of 2018 legislated that workers should work an average of 40 h per week, and overtime working hours should not exceed 12 h. However, the law does not protect self-employed workers and non-regular workers and prevent overworked situations [7, 8].

Long working hours may lead to health problems such as fatigue [9], headache [10], myocardial infarction [11], coronary heart disease [12, 13], and cardiac disease [14]. Furthermore, some studies have documented that overworking may increase both all-cause and specific disease-related mortality [15–17]. For example, O’Reilly and Rosato showed that men in routine or semi-routine occupations who worked more than 55 h per week had a 31% higher risk of all-cause mortality than men who worked 35–40 h per week [18].

Long working hours may modify workers’ lifestyles. Working hours have an intimate relationship with sleep conditions; the longer the working hours, the shorter the absolute sleep duration per day. Long working hours may also cause sleep disturbance, which negatively affects mental health and recovery from diseases [13]. In addition, reduced sleep duration due to the stress related to overwork may contribute to poor health behaviors such as smoking and alcohol drinking [19]. Given the imbalance between the sleep cycle and work-life, long working hours may have a negative impact on general well-being [20].

Self-rated health (SRH) has been widely used as an indicator that evaluates an individual’s health because it reflects the general state of disease and health condition [21]. Prior studies have found evidence on the relationship between working hours and SRH across contexts. Park [22] showed that male workers who worked 53–60 h per week reported poorer SRH than those who worked 36–40 h (OR: 1.683, 95% CI: 1.02, 2.77), while Ryu [23] showed that men who work less than 40 h per week were less likely to have poorer health outcomes than those who work 40–46 h (prevalence ratio: 0.89, 95% CI: 0.86, 0.91).

The World Health Organization defines quality of life (QOL) as “individuals’ perceptions of their position in life in the context of the culture and value systems in which they live and in relation to their goals, expectations, standards and concerns” [24]. QOL measurements include multi-dimensional aspects of life quality, and health-related QOL (HRQOL) assesses comprehensive aspects of physical and mental health status [25]. Thus, monitoring the HRQOL score promotes people’s awareness of their health behaviors and risk factors, which in turn helps prevent various health-related problems [26].

Some previous studies have identified the following relationships separately: working hours-sleep duration [27], sleep duration-HRQOL [28, 29], working hours-poor SRH [22, 30], and poor SRH-HRQOL [31, 32]. However, only a few studies have investigated the relationships between working hours, sleep duration, SRH, and HRQOL simultaneously [19, 20]. Furthermore, most these studies focused on a single population, such as nurses [21] or white-collar professionals [22]. No studies have examined the nature of the relationships between working hours, sleep duration, SRH, and HRQOL among the general male workers in Korea. The aim of this study was to investigate whether and how working hours are associated with the HRQOL of Korean male population workers. We examined the contributions of sleep duration and poor SRH to explaining the link between working hours and HRQOL using a nationally representative sample. We hypothesized that sleep duration and poor SRH would be mediators in the relationship between working hours and HRQOL.

## Methods

### Study population

For this study, we used data from the 2016–2017 waves of the Korea National Health and Nutrition Examination Survey (KNHANES). This survey, which is conducted by the Korea Centers for Disease Control, entails 23 households extracted from each of 192 districts each year and assesses household members who are over 1 year old. The KNHANES employed a complex sampling design that involves stratified and multistage probability clustering. The detailed survey design and methods have been described elsewhere [33]. Among the participants aged 1 to 80 years (*n* = 16,277), we used data from male participants aged 20 to 49 years old (*n* = 2650). We excluded 509 participants who reported missing values ​​for any of the following major variables: working hours, sleep duration, HRQOL, or poor SRH. Ultimately, we included 2141 men as the final study participants.

### Measurements

Measurements of predictor, mediator and outcome were collected by the systematic health interview which has been conducted by trained interviewers using a standardized health questionnaire. The health interview questionnaire included information on working hours, sleep duration, SRH, and HRQOL, which are collected by self-administration. The survey estimates working hours, our predictor of interest, for individuals who worked more than one hour for income purposes or more than 18 h for unpaid family work in the previous week with the following question: “How many hours did you work on average last week at a job?” The variables hypothesized to be mediators were sleep duration and poor SRH. Sleep duration was calculated as the interval between sleeping and waking time on a weekday, and SRH was measured with the question “How do you feel about your overall health condition?” The item was rated on a five-point scale with responses divided into two categories: normal (very good, good, or fair) or poor (poor or very poor) health. Our outcome variable of interest was HRQOL, which was evaluated using the EuroQol-5 Dimension (EQ-5D); the five dimensions of the EuroQol-5 are mobility, self-care, usual activity, pain/discomfort, and anxiety/depression. The EQ-5D index score ranges from − 0.171 to 1, with 1 indicating no problem in any of the five dimensions, 0 indicating death, and a negative value indicating poorer health than death [34]. In this study, we multiplied the measured EQ-5D score by 100, and the range of the converted scores was thus − 17.1 to 100.

### Covariate measures

The following covariates were taken into account in this study: income (lowest, lower middle, upper middle, or highest), education (middle school or below, high school, or college or above), marital status (single or married), occupation (white collar or professional, service sales, blue collar), smoking (current smoker or ex-smoker or non-smoker), alcohol drinking (≥1 time a week or none and < 1 time a week), hypertension (systolic blood pressure ≥ 140 mmHg and diastolic blood pressure ≥ 90 mmHg or current treatment), diabetes (glucose ≥126 mg/dL or current treatment), and obesity (body mass index ≥25 kg/m^2^).

### Statistical analysis

We analyzed the data on general characteristics in men, and our data were presented as the estimated proportion (standard error, SE) for discrete variables and the estimated mean (SE) for continuous variables. We used four multivariable linear regression models to investigate the relationship between working hours and HRQOL. Model 1 focused on the relationship between working hours (unit: 10 h/w) and HRQOL without any adjustment. In Model 2, we adjusted for age; in Model 3, we further adjusted for income, education, occupation, marital status, smoking, alcohol drinking, hypertension, diabetes mellitus, and obesity; and in Model 4, we further controlled for sleep duration and poor SRH. We performed a mediation analysis to investigate the contributions of sleep duration and poor SRH to the association between working hours and HRQOL. We used Mplus 8 (Muthen and Muthen, 1998–2017) for the mediation analysis and SAS 9.4 (SAS Institute, Cary, NC, USA) for all other analyses. We considered statistical significance at *P value* < 0.05.

### Ethics

Our study was exempt from the institutional ethical review board at the University of Seoul (IRB No. 2019–24).

## Results

Our study population was comprised of 2141 men (Table 1) with a mean age of 35.7 years. Most of the participants had a university education or higher, and most were considered white collar or professional workers. More than half of the participants were married and drank more than once a week. The participants worked a mean of 45.5 h a week and slept a mean of 6.9 h per day. A total of 11.7% of the participants experienced poor SRH conditions. The mean HRQOL summary score was 98.3. In particular, 10.9% of men experienced a problem in the pain/discomfort components of HRQOL, which was the highest problem rate among HRQOL components. Conversely, the HRQOL component with the lowest problem rate was self-care, in which 0.4% of participants reported such a problem.

**Table 1:** Characteristics of study participants

Variables	N	Mean (SE) or Proportion (SE)
Age, y	2141	35.7 (0.2)
Household income (quartile), %		
Lowest	488	24.2 (1.2)
Lower middle	588	25.8 (1.1)
Upper middle	552	25.5 (1.1)
Highest	542	24.4 (1.2)
Education level, %		
Middle school or below	80	3.5 (0.5)
High school	749	37.5 (1.4)
College or above	1312	59.0 (1.5)
Occupation, %		
White collar/professional	1020	51.2 (1.5)
Service sales	293	15.5 (1.0)
Blue collar	643	33.3 (1.5)
Marital status, married, %	1400	59.4 (1.6)
Smoking, %	962	45.2 (1.3)
Alcohol drinking, %	1711	79.9 (1.0)
Hypertension, %	429	19.5 (0.9)
Diabetes mellitus, %	429	4.8 (0.6)
Obesity, %	957	44.2 (1.2)
Working hours, h/w	2141	45.5 (0.4)
Sleep duration, h/d	2141	6.9 (0.0)
Poor SRH, %	230	11.7 (0.8)
HRQOL, score	2141	98.3 (0.1)
Problem in HRQOL components, %		
Mobility	55	2.6 (0.4)
Self-care	11	0.4 (0.1)
Usual activity	30	1.4 (0.3)
Pain/discomfort	233	10.9 (0.7)
Anxiety/depression	88	4.1 (0.5)

Data are expressed as mean (SE) or proportion (SE). Each component of HRQOL was rated on two level with responses divided into two categories: no problems versus some/extreme problems

Without any adjustments, working hours were significantly associated with HRQOL in men [Model 1; coefficient: − 0.238; 95% confidence interval (CI): − 0.431, − 0.044] (Table 2). After we adjusted for age, the relationship between working hours and HRQOL remained statistically significant (Model 2; coefficient﻿: − 0.257; 95% CI: − 0.463, − 0.052). When socioeconomic status, hypertension, diabetes mellitus, obesity, and health behaviors were added, the strength of the relationship did not decrease (Model 3; coefficient﻿: − 0.254; 95% CI: − 0.431, − 0.077). After we added sleep duration and poor SRH (Model 4; coefficient﻿: − 0.199; 95% CI: − 0.360, − 0.037), the strength of the relationship between working hours and HRQOL significantly decreased. This result indicates that these two variables (sleep duration and poor SRH) might contribute to explaining the association between working hours and HRQOL.

**Table 2:** The relationship between working hours (unit: 10 h/w) and HRQOL

Model	Coefficient (95% CI)	*P* value
1	−0.238 (−0.431, −0.044)	0.016
2	−0.257 (−0.463, −0.052)	0.014
3	−0.254 (−0.431, −0.077)	0.005
4	−0.199 (−0.360, −0.037)	0.016

Model 1: unadjusted. Model 2: adjusted for age. Model 3: further adjusted for income, education, occupation, marital status, smoking, alcohol drinking, hypertension, diabetes mellitus, and obesity. Model 4: further adjusted for sleep duration and poor SRH

**Fig. 1:**
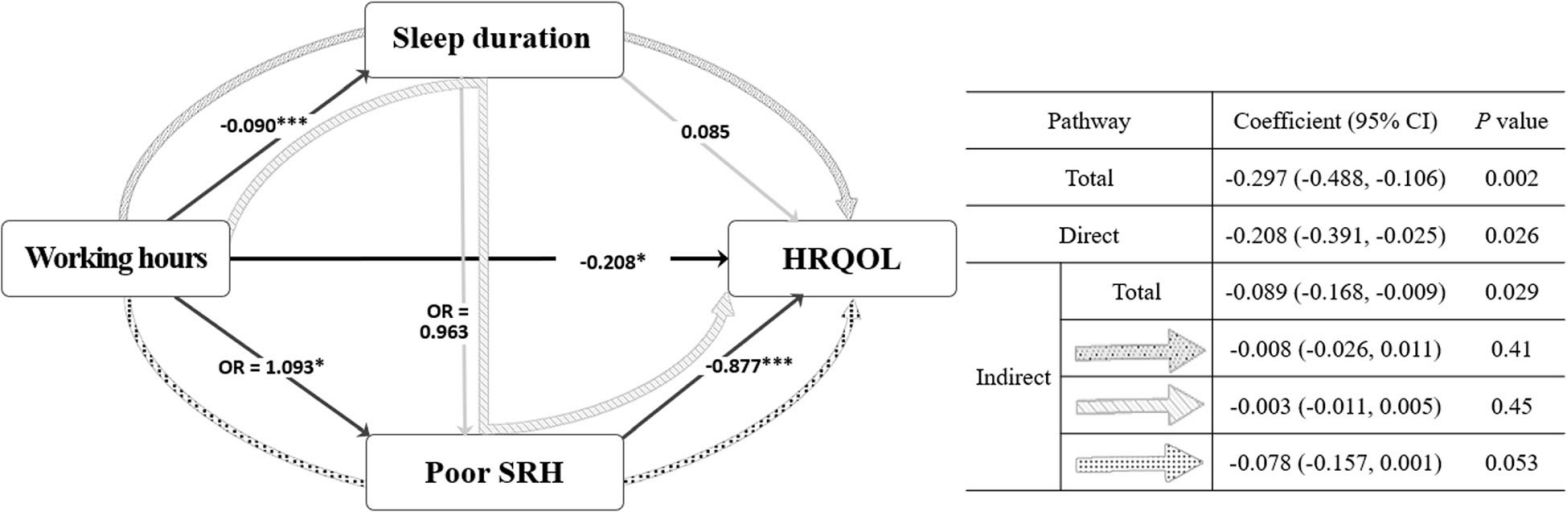
The relationship between working hours (unit: 10 h/w), sleep duration (unit:1 h/d), poor SRH, and HRQOL. Each model adjusted for the following variables: age, income, education, occupation, marital status, smoking, alcohol drinking, hypertension, diabetes mellitus, and obesity (*P value < 0.05, **P value < 0.01, and ***P value < 0.001)

In the mediation analysis, the total effect of working hours on HRQOL was statistically significant (coefficient﻿: − 0.297; 95% CI: − 0.488, − 0.106; Fig. 1). Working hours directly influenced HRQOL, and poor SRH significantly mediated this association while sleep duration barely explained the relationship; the estimated mediation effect was 26.3%. Working hours were strongly associated with the following three HRQOL indicators: self-care, usual activity, and pain/discomfort (Table 3). Among these three factors, working hours had significant total and indirect impacts on self-care and had total, direct, and indirect impacts on usual activity.

**Table 3:** Mediation analysis for the relationship between working hours (unit: 10 h/w) and any problem of HRQOL components through sleep duration and poor SRH

HRQOL components	OR (95% CI)	*P* value
Mobility		
Total effect	1.039 (0.934, 1.156)	0.48
Direct effect	1.025 (0.925, 1.135)	0.64
Indirect effect	1.013 (0.988, 1.039)	0.30
Self-care		
Total effect	1.090 (1.008, 1.179)	0.030
Direct effect	1.047 (0.962, 1.140)	0.29
Indirect effect	1.041 (1.001, 1.083)	0.045
Usual activity		
Total effect	1.195 (1.090, 1.311)	0.000
Direct effect	1.130 (1.041, 1.228)	0.004
Indirect effect	1.058 (1.008, 1.108)	0.022
Pain/discomfort		
Total effect	1.114 (1.027, 1.208)	0.009
Direct effect	1.077 (0.995, 1.165)	0.07
Indirect effect	1.035 (0.994, 1.077)	0.10
Anxiety/depression		
Total effect	1.105 (0.944, 1.294)	0.21
Direct effect	1.052 (0.890, 1.245)	0.55
Indirect effect	1.050 (1.001, 1.102)	0.045

*OR* odds ratio. Each model adjusted for the following variables: age, income, education, occupation, marital status, smoking, alcohol drinking, hypertension, diabetes mellitus, and obesity

## Discussion

Based on the results of previous studies, we expected to identify strong relationships between working hours and HRQOL that were partly mediated by sleep duration and SRH. The current study found that longer working hours were associated with poorer HRQOL in men and that long working hours affected HRQOL through poor SRH. Men with longer working hours were more likely to have problems with self-care, usual physical activities, and pain/discomfort.

The current study documented that working hours strongly influenced poor SRH. Longer working hours appeared to increase exposure to dangerous and stressful work environments [35], and thus, men who overwork may perceive themselves as unhealthier than others [36]. It is also plausible that people who work longer hours are less likely to exercise regularly [19, 37] and more likely to eat unhealthy foods. Longer working hours may lead to poor health behaviors such as smoking, alcohol drinking, and a sedentary lifestyle, which determine individuals’ health perception [38]. SRH has a major impact on HRQOL given that subjective health perception shapes QOL [39].

The current study found that working hours were significantly related to sleep duration; however, sleep duration did not explain the relationship between working hours and HRQOL. The result implies that there may be unexplained pathways underlying the link between working hours and HRQOL other than sleep duration.

The direct association between working hours-HRQOL was still significant even after controlling for SRH and sleep duration, which indicates that there may be unexplored mechanisms other than these factors. We speculated that poor HRQOL may be directly attributable to overwork-related stressors that cannot be properly managed by coping strategies [40]. In addition, fatigue and obesity due to the lack of physical activity and self-care may lead to functional problems, which negatively affects HRQOL.

However, this study has several strengths. First, this study is the first to identify the pathway underlying the relationship between working hours and HRQOL by directly testing the mediation effect of sleep duration and poor SRH. Furthermore, this study used data obtained from a nationally representative sample that included a wide range of male workers such as part-time, self-employed, and unpaid family workers. Thus, our findings may reflect characteristics of a broader scale of workers who had not been investigated in previous studies.

The current research has several limitations. First, the data on working hours and sleep duration were self-reported, and thus there may be recall bias (i.e., inaccurate recollections). Second, because the survey was a cross-sectional study, the causality of the relationship between working hours and HRQOL could be overstated. Longitudinal studies are necessary to clarify the relationship between working hours, SRH, and HRQOL. Lastly, given that our study sample is from Korea, future studies must consider how these results can be generalized to populations in different contexts.

## Conclusions

This study demonstrated a significant relationship between working hours and HRQOL; the effects was significantly mediated by poor SRH. Our finding suggests that reducing working hours and improving work-life balance can promote men’s health and QOL, regardless of their type of job. Longitudinal data are necessary to investigate the variables that mediate this relationship.

## Data Availability

The datasets supporting our study findings are available in the Korea Centers for Disease Control and Prevention (https://knhanes.cdc.go.kr/knhanes).

## Abbreviations

EQ-5D: EuroQol-5 Dimension
HRQOL: Health-related quality of life
KNHANES: Korea National Health and Nutrition Examination Survey
QOL: Quality of life
SRH: Self-rated health
